# Supplementary material for: Bioinformatic identification of ClpI, a distinct class of Clp unfoldases in Actinomycetota
Source: Front Microbiol. 2023 Apr 17;14:1161764. doi: 10.3389/fmicb.2023.1161764 (PMC10149685; doi:10.3389/fmicb.2023.1161764)
Supplement: Supplementary file 1 [file Data_Sheet_1.PDF]

Table S1. Key residues that differ among ClpC/ClpB/ClpI enzymes.

B. subtilis ClpC	*M. tuberculosis* ClpC1	Actinomycetota ClpC	Actinomycetota ClpB	Actinomycetota ClpI
S57	L56	L	P/Q	P
T105	T105	T	T	P
T160	S168	S	K	T
D169	N177	N	D	D
R191	R199	R	R	Q
E194	Q202	Q	Q	E
N298	S306	S	N	N
Q310*	Q318	Q	R	H/R
E397	E405	E	E	Q
V431	I439	I	Q/E	V
Q434	Q442	Q	G	E
E446	E454	E	E	I
M502	M510	M	M/L	L
I542	I550	I	L	L
L544	A552	A	L	L
G599	G607	G	G	A
Y611	F619	F	Y	Y
E707*	D715	D	D/E	E
H712*	H720	H/P	D/S/H	R/H
E774*	E782	E	G	D

* Noted as differing among proteobacterial ClpA/E/L/C proteins (Miller et al., 2018).

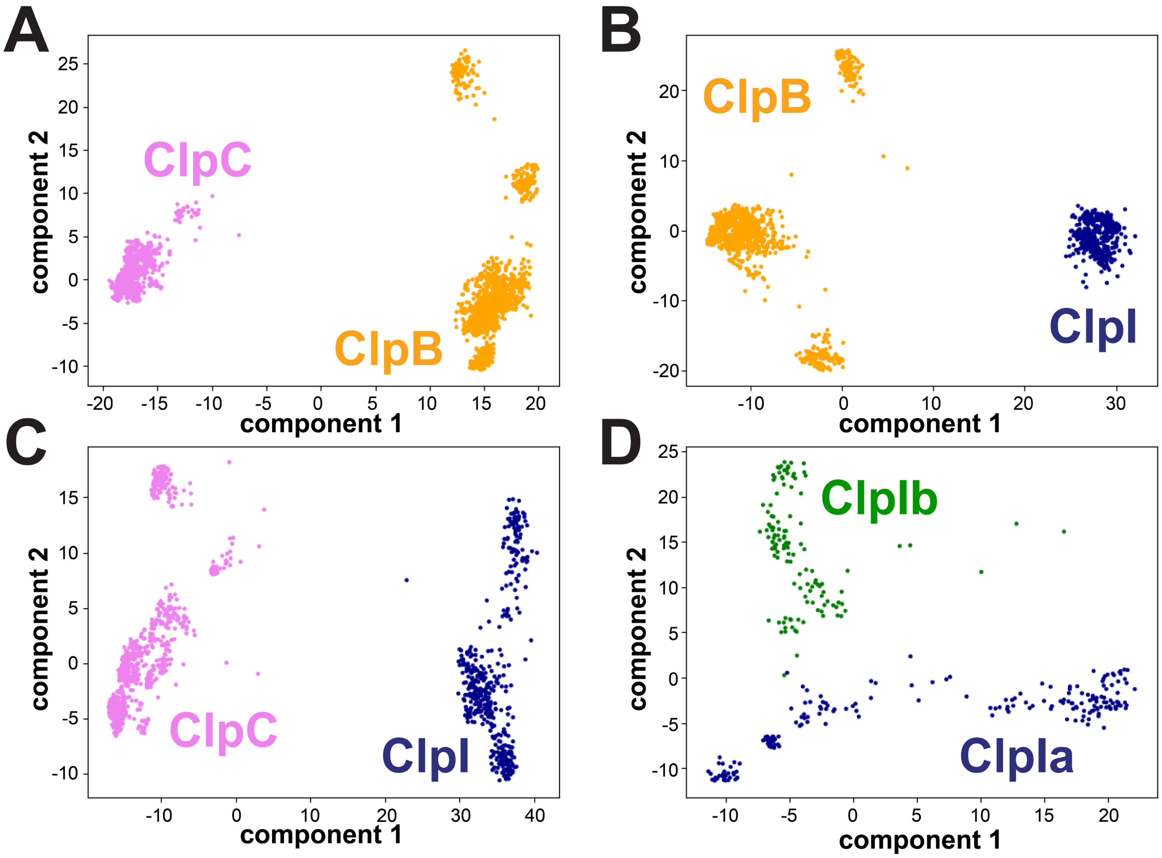

Figure S1. Principal component analysis. Clp enzyme orthologs from Actinomycetota were subjected to PCA analysis, comparing (**A**) ClpC (violet) and ClpB (orange), (**B**) ClpB (orange) and ClpI (blue), (**C**) ClpC and ClpI, or (**D**) ClpIa (blue) and ClpIb (green).

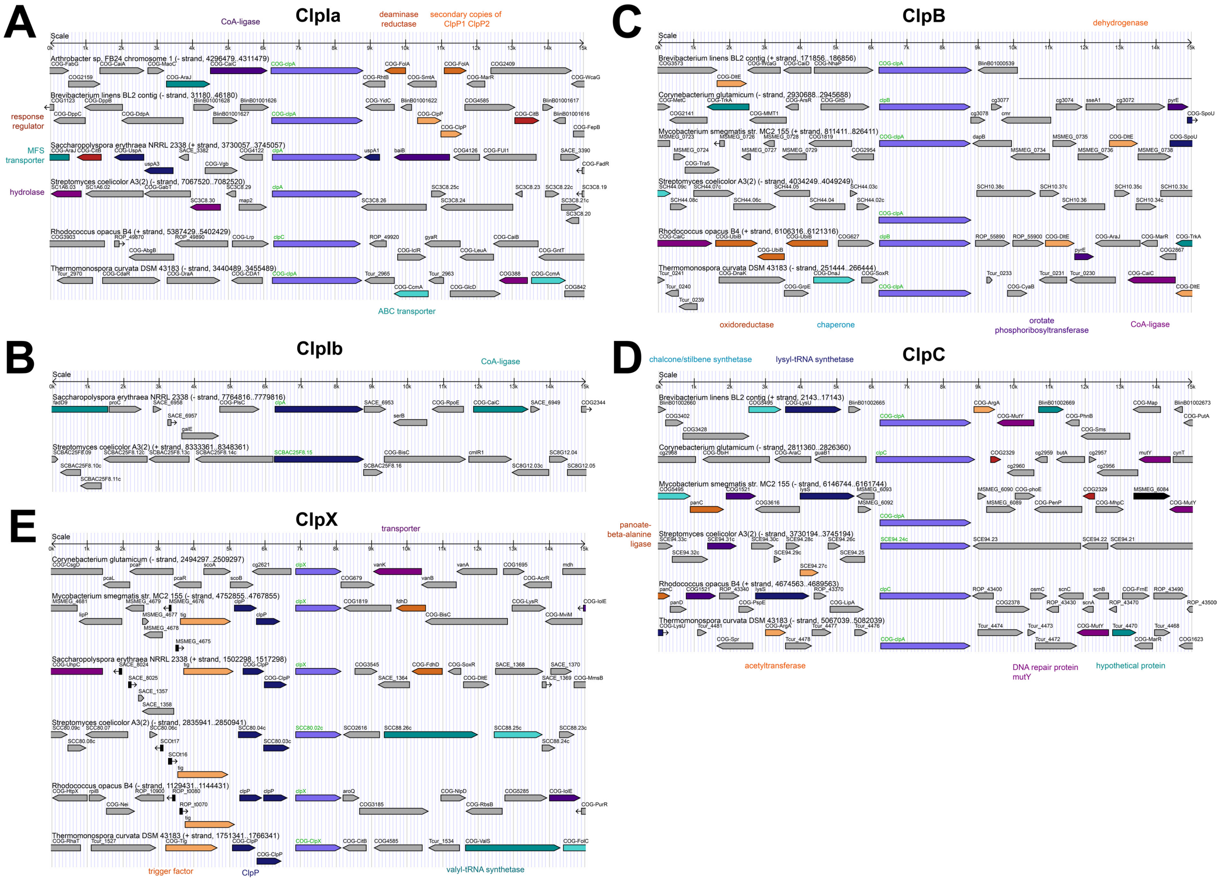

Figure S2. Genomic context of unfoldase genes. Flanking genomic regions surrounding representative **A**) *clpIa*, **B**) *clpIb*, **C**) *clpB*, **D**) *clpC*, and **E**) *clpX* genes were generated using the MicrobesOnline resource (Dehal et al., 2010). Recurring genes with notable sequence homology are colored and annotated in the margins surrounding the figures.

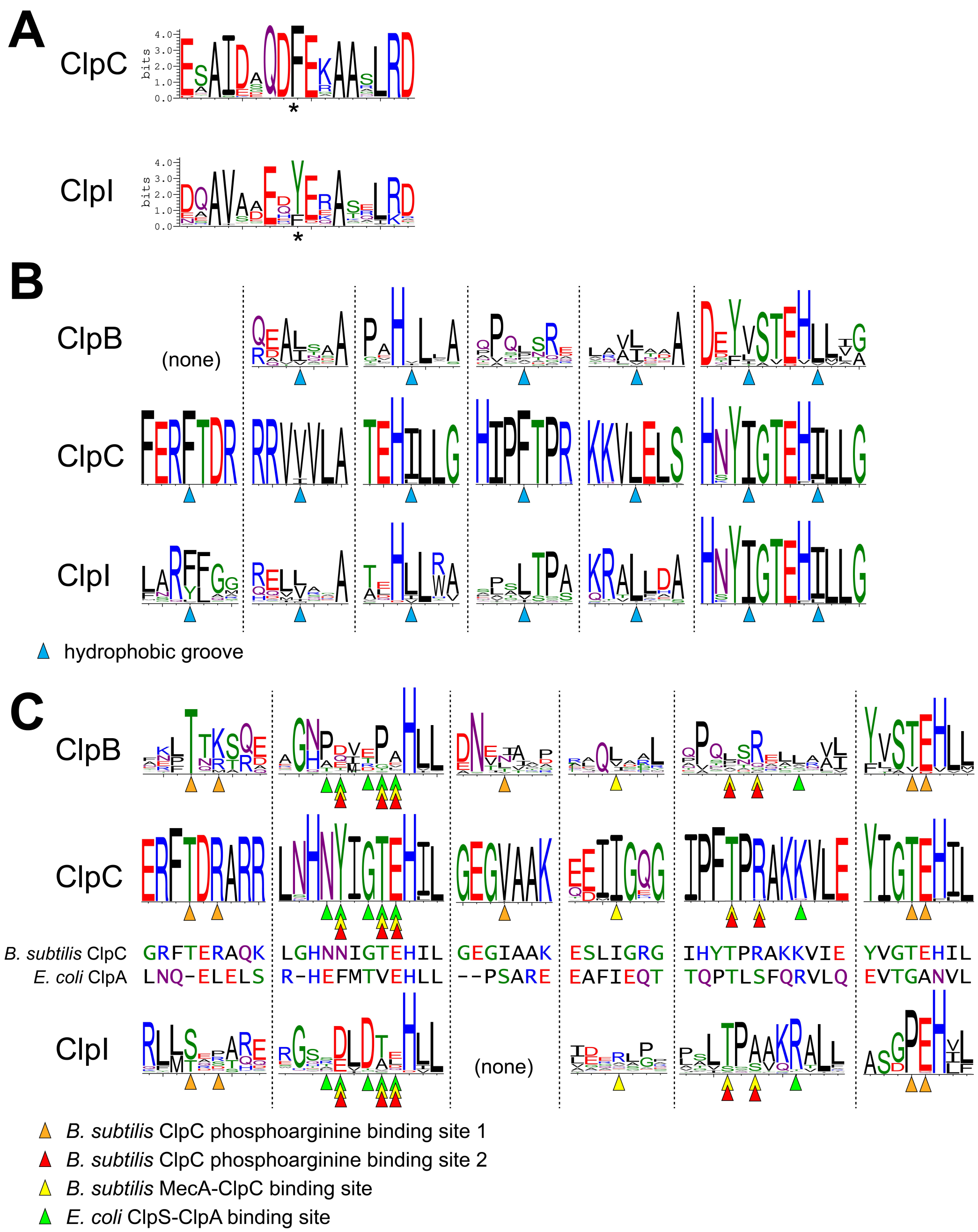

Figure S3. Sequence conservation patterns of key motifs. **A**) Sequence logos show the M-domain tip region in ClpC and ClpI orthologs. The asterisk marks the hydrophobic residue at the M-domain tip. **B**) Sequence logos show regions surrounding the residues that form the hydrophobic groove (blue arrow) (Li and Sha, 2003; Rosenzweig et al., 2015; Rizo et al., 2019). **C**) Sequence logos show regions involved in binding of phosphoarginine (Fuhrmann et al., 2009; Weinhäupl et al., 2018; Ogbonna et al., 2022) or MecA (Wang et al., 2011) to ClpC in *B. subtilis* or ClpS to ClpA in *E. coli* (Guo et al., 2002; Zeth et al., 2002).

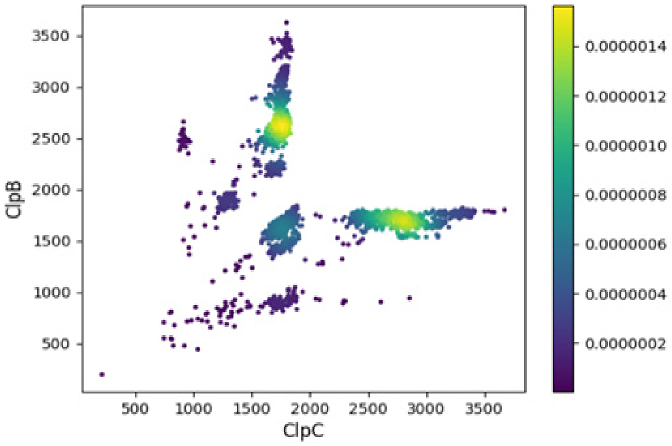

Figure S4. BLAST analysis of Actinomycetota ClpB/C orthologs omitting NTD. The NTD region was removed from ClpC/B orthologs, and the resulting sequences were compared to *Mycobacterium smegmatis* ClpB (A0QQF0_MYCS2) and ClpC (CLPC1_MYCS2) references as in **Figure 2**. The *x* axis represents the BLAST score against the ClpC reference, and the *y* axis represents the BLAST score against the ClpB reference.

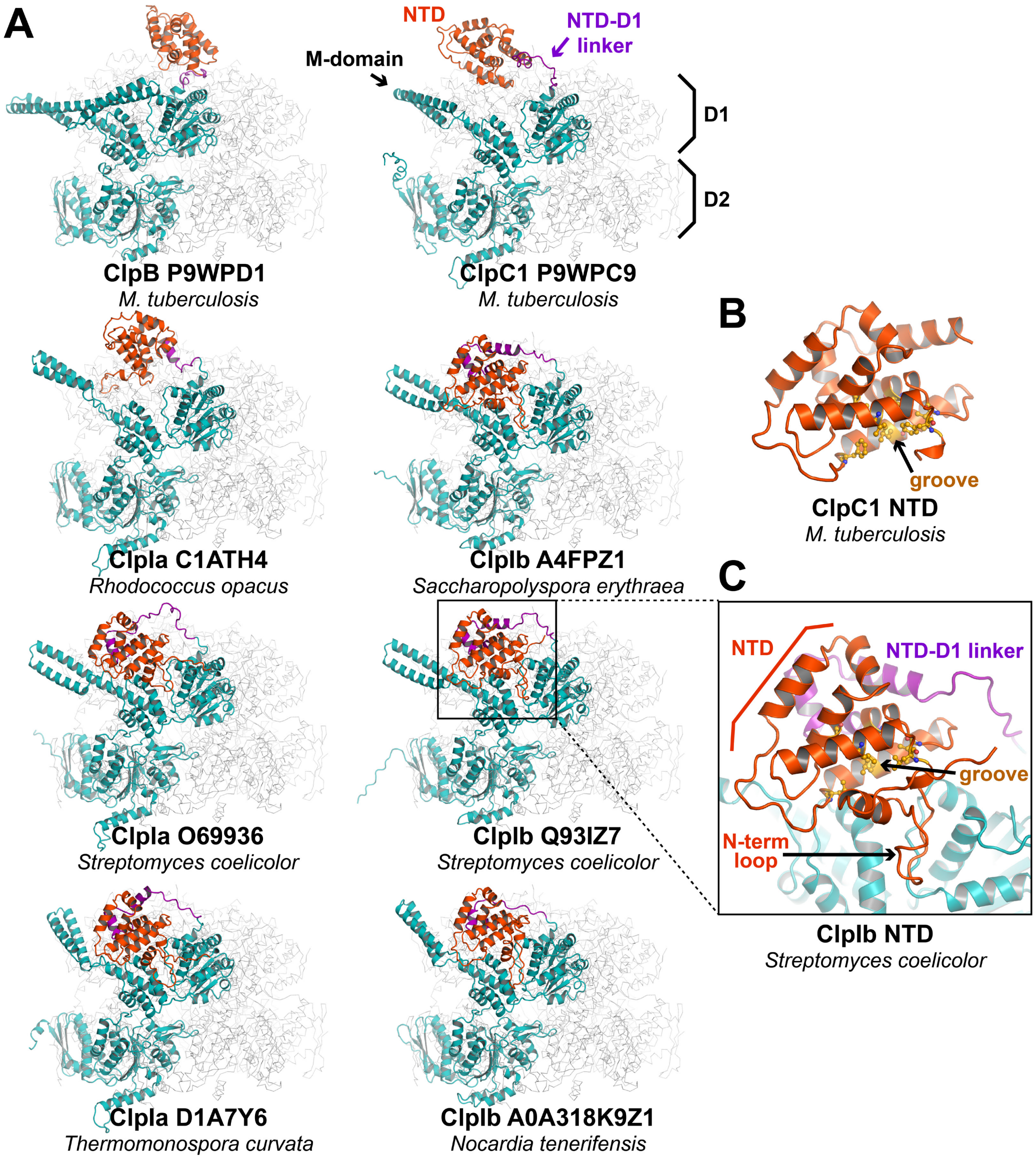

Figure S5. AlphaFold models of the NTD and linker region. A) The indicated AlphaFold models (AF accession IDs shown; Jumper et al., 2021) are represented as teal cartoons with an orange NTD and purple NTD-D1 linker region. Models are aligned to one subunit of a ClpC1 hexamer from (gray ribbons; PDB ID: 8A8W) for reference. **B**) Detail of the ClpC1 NTD from AlphaFold model P9WPC9. Residues forming the hydrophobic groove are shown as gold ball-and-sticks. **C**) Detail of the *Streptomyces coelicolor* ClpIb NTD from AlphaFold model Q93IZ7, with hydrophobic groove residues colored as in B.
